# A targeted DNA substrate mechanism for the inhibition of HIV‐1 integrase by inhibitors with antiretroviral activity

**DOI:** 10.1002/2211-5463.12025

**Published:** 2016-02-24

**Authors:** Farah F. Ammar, Zeina Hobaika, Safwat Abdel‐Azeim, Loussinée Zargarian, Richard G. Maroun, Serge Fermandjian

**Affiliations:** ^1^ Centre d'Analyses et de RechercheUR EGFEMFaculté des SciencesUniversité Saint‐JosephBeirutLebanon; ^2^ LBPA, UMR8113 du CNRSEcole Normale Supérieure de CachanCedex CachanFrance; ^3^ Chemistry and Biology, Nucleo(s)tides and Immunology for TherapyUMR8601 CNRSParis Cedex 06France

**Keywords:** DNA, HIV‐1, inhibitors, integrase, interaction, intercalation

## Abstract

We recently reported that viral DNA could be the primary target of raltegravir (RAL), an efficient anti‐HIV‐1 drug, which acts by inhibiting integrase. To elucidate this mechanism, we conducted a comparative analysis of RAL and TB11, a diketoacid abandoned as an anti‐HIV‐1 drug for its weak efficiency and marked toxicity, and tested the effects of the catalytic cofactor Mg^2+^ (5 mm) on drug‐binding properties. We used circular dichroism and fluorescence to determine drug affinities for viral DNA long terminal repeats (LTRs) and peptides derived from the integrase active site and DNA retardation assays to assess drug intercalation into DNA base pairs. We found that RAL bound more tightly to LTR ends than did TB11 (a diketo acid bearing an azido group) and that Mg^2+^ significantly increased the affinity of both RAL and TB11. We also observed a good relationship between drug binding with processed LTR and strand transfer inhibition. This unusual type of inhibition was caused by Mg^2+^‐assisted binding of drugs to DNA substrate, rather than to enzyme. Notably, while RAL bound exclusively to the cleavable/cleaved site, TB11 further intercalated into DNA base pairs and interacted with the integrase‐derived peptides. These unwanted binding sites explain the weaker bioavailability and higher toxicity of TB11 compared with the more effective RAL.

**Abbreviations**
3′Pr: 3′processing
CD: circular dichroism
DKA: diketoacid
DTG: dolutegravir
EVG: elvitegravir
IN: integrase
INSTI: integrase strand transfer inhibitor
RAL: raltegravir
ST: strand transfer

The human imunodeficiency virus type I (HIV‐1) is known as the causative agent of AIDS 1. Integrase (IN) 2, encoded by the virus pol gene, catalyses integration sequentially through the deletion of two nucleotides at the conserved 3′ extremity of the viral long terminal repeats (LTRs) [3′ processing (3′Pr)] and the covalent insertion of the recessed 3′ viral DNA into the cell genome [strand transfer (ST)] 3, 4, 5. The catalytic domain of HIV‐1 IN bears the active site acidic residues, Asp64, Asp116 and Glu152 (the so‐called DDE catalytic triad). Each of these residues is critical for 3′Pr, ST and disintegration reactions 6.

HIV‐1 IN has attracted considerable attention as a potential drug target, as it has a key role in the virus life cycle and has no cellular counterpart 7. As expected, the introduction of IN inhibitors into antiretroviral therapy has contributed significantly to limiting the spread of AIDS. The three approved IN inhibitors, raltegravir (RAL, MK‐0518, Isenstress^®^ from Merck and Co, Inc., Whitehouse Station, NJ, USA), elvitegravir (EVG, JTK‐303/GS‐9137 from Japan Tobacco Inc. and Gilead Sciences, Foster City, CA, USA) 8, 9 and dolutegravir, (DTG, S/GSK‐1349572 or ‘572’, Tivicay by Glaxo Smith Kline, Research Triangle Park, NC, USA) 10 have only minor resistance mutations and belong to a class of IN inhibitors that act on ST (INSTIs), although they can also affect 3′Pr to a certain extent 11. Chemically, they derive from or are isosteres of diketoacids (DKA), a class of compounds originally designed to chelate divalent cations and mediate the association of the negatively charged carboxylate and phosphate groups of the substrate DNA and serve as cofactors in the catalysis 12.

For many years, the cocrystal of 1‐(5‐ChloroIndol‐3‐yl)‐3‐hydroxy‐3‐(2H‐TEtrazol‐5‐yl)‐Propenone (5CITEP) (Table 1) binding to the catalytic core domain (CCD) of HIV‐1 IN was the only available structure of an inhibitor bound to a key domain of the enzyme 13. 5CITEP shares six interactions with each CCD monomer, five of which have the α4 helix. Of note, in the cocrystal 5CITEP‐CCD complex, we do not observe chelated divalent cations. The carboxylic group of the key Glu152 residue located at the beginning of the α4 helix establishes direct interactions with the diketo motif 13. This structure has raised numerous questions on the functional location of INSTIs and the role of divalent metal ions during inhibition. Actually, the calculated models of viral DNA‐IN complexes have invariably placed INSTIs within the cavity formed by the active site and the processed LTR end, with the diketo (or equivalent) motif engaged in interactions with a pair of divalent metal cations 14, 15. It was only recently that x‐ray structures of RAL (Table 1) and several other INSTIs in complex with the intasome that is, the complex of IN with both processed and unprocessed DNAs, have been solved 16, 17, 18, thanks to the use of the highly soluble and robust PFV (prototype foamy virus) enzyme 19. Regardless of the inhibitor, the cocrystal structure showed the coplanar oxygen (or nitrogen) motif of INSTIs at a chelating distance from a divalent ion pair fixed to the DNA end, and the halobenzyl group within van der Waals interactions with the highly conserved 5′CA 3′/5′TG 3′ dinucleotide step 16, 17, 18, 20, 21.

**Table 1:** Three compounds illustrating the improvement of IN inhibition and antiviral efficiency during the last decade are indicated: the chemical structures, the *in vitro* IC50, the antiviral efficiencies and the article references for the 5CITEP, TB11 and RAL

Compound	Structure	Reaction	*In vitro* IC50 (μm)	Antiviral	References
5CITEP	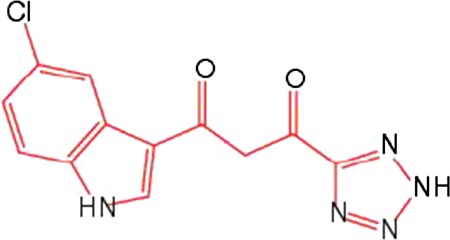	ST	97 ± 32	No	12, 13
		3′Pr	400		
		3′Pr/ST	≈ 4		
TB11	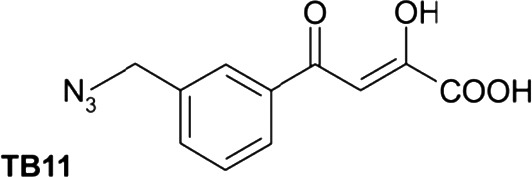	ST	0.33 ± 0.03	Yes but bad bioavailability	12
		3′Pr	70.9/> 100		
		3′Pr/ST	≈ 300		
RAL	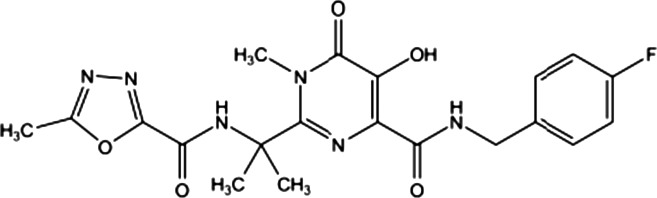	ST	0.002–0.007	FDA approved Oct. 2007	90, 91
		3′Pr	34 ± 3		
		3′Pr/ST	≈ 1270		

The x‐ray studies devoted to the binding of INSTIs to the intasome elucidated the mechanism of action of potent drugs and the origin of resistance mutations in the HIV‐1 enzyme 16, 17, 20, 21, 22, 23, 24. However, studies performed in solution have demonstrated the inability of INSTIs to bind the enzyme or CCD alone 25, 26, in contrast to 5CITEP 13. This prompted us to examine the possible role of DNA alone in drug binding in our recent work 27. In the latter, we showed that RAL was able to tightly bind to the free unprocessed and processed LTR ends. Furthermore, the affinity of RAL for the processed LTR end alone appeared greater compared with that reported for the intasome 27, 28, thereby suggesting that the substrate DNA could be the primary target of INSTIs. However, in previous work, we did not examine the crucial role played by Mg^2+^ ions in the stabilization of the drug‐DNA complex. Mg^2+^ is involved in bridging electrostatic interactions between the co‐planar oxygens of the drug 29, 30 and selected DNA phosphate oxygens 31, 32, 33.

We initiated this study from the idea that TB11 and RAL, which largely differ in their chemical structure and efficacy (Table 1), could have different binding sites. The TB11 structure somewhat resembles that of 5CITEP, but its efficiency towards HIV‐1 infection is considerably greater 12. Much is already known about the binding and structural properties of RAL, but there was a lack of physicochemical data on TB11. In prior studies, hope was placed on TB11 as a possible antiviral drug 12, 34, but it was abandoned because of its low oral bioavailability and toxicity. In contrast to TB11, RAL did not have the same adverse effects. It has dose limiting side effects (50% cytotoxic concentration ~ 60–600 μm) 35 and displays a marked bioavailability. Thus, it was questioned whether TB11 in the range of the high concentrations used had additional targets besides the substrate virus that were responsible for the side effects 8, 29.

In this study, we used circular dichroism (CD) and fluorescence (anisotropy) to evaluate the RAL and TB11 binding affinities for: (a) peptides derived from the IN active site (K156 and K156‐*loop140*) and (b) oligonucleotides mimicking processed and unprocessed substrates (LTR32 and LTR34) and other structural analogues. We also performed plasmid gel retardation and unwinding assays to test the ability of drugs to intercalate into the DNA base pairs.

## Materials and methods

### Oligonucleotides

**Figure 1:**
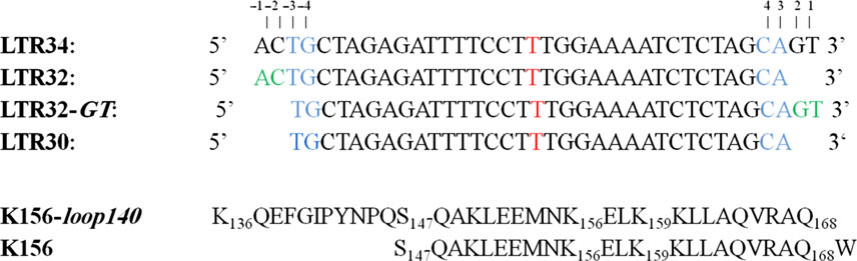
The oligonucleotides and the peptides used in this work.

These were purchased from Eurogentec (Liège, Belgium). All are single stranded oligonucleotides (Fig. 1) designed to adopt a folded double‐stranded hairpin structure, especially at the low concentrations required in fluorescence spectroscopy 36, 37. Fluorescence studies were possible thanks to the fluoresceine fluorophore grafted on the thymine (in red) at the centre of the added 3T forming a loop. The so‐called LTR34 folds into a 17‐base pair stem, which mimics the unprocessed U5 LTR end of viral DNA. The folded LTR32 mimics the processed U5 LTR end. It has a stem with 15 base pairs and bears a dinucleotide AC overhang (in green) at the 5′ terminus of the nontransferred strand. LTR32‐*GT* with an added GT overhang (in green) at the 3′ end and the blunt‐ended LTR30 lacking the two cleavable base pairs 5′GT3′:5′AC3′ were designed to assess the impact of the terminal dinucleotides on binding. In the four oligonucleotides, the highly conserved base pairs 5′CA3′/5′GT3′ are coloured in blue.

### Peptides K156 and K156‐loop 140

These two peptides (Fig. 1), were synthetized by Christophe Piesse at the University Pierre et Marie Curie to a purity grade > 98%. The K156 peptide 36, 37 that contains the residues 147–169 includes a stabilized version of the α4 helix (residues 150–166) found in the HIV IN CCD crystal by Maignan *et al*. 38. This α4 helix possesses an additional turn at its N terminus compared with that of Dyda *et al*. 39, and is superimposable with the α4 helix of the avian sarcoma virus IN CCD 40. The K156‐*loop140* peptide (residue 136 to residue 168) comprises the modified α4 helix portion and the native unmodified flexible loop140 at its N terminus. The peptide concentration was estimated for K156‐*loop140* from the UV signal intensity of the intrinsic tyrosine occupying position 143 in IN and for K156 from the signal of tryptophan added at the C‐terminal position for this purpose, using a molar absorption coefficient at 280 nm of 1280 and 5600 m ^−1^·cm^−1^ respectively.

### Inhibitors

RAL 8 is produced by Merck & Co. and was purchased from CacheSyn Inc. (Mississauga, ON, Canada). TB11 (MA‐DKA) was synthetized by the Burke's group (NCI) 30, 34. The powders are soluble in water. The structures of RAL and TB11 are shown in Table 1.

### CD measurements

CD spectra between 190 and 260 nm (peptides and peptide‐drug complexes) and between 190 and 330 nm (DNA and peptide‐DNA complexes) were recorded with a Jobin‐Yvon CD6 dichrograph using a quartz cell of 1 mm path length. Measurements were calibrated with (+)‐10‐camphosulfonic acid. Spectra of samples dissolved in phosphate buffer (10 mm, pH 6.0) with 5 mm Mg^2+^ were recorded in 1‐nm steps, corrected for the base line, and averaged over 10 scans. Before spectral recording, samples were incubated for 10 min at the chosen temperature to allow the solutions to reach their equilibrium state. For titration experiments desired ratios were obtained by addition of the ligand (generally 1–20 μm) to the compound kept at a constant amount (generally 20 μm) in the thermally jacketed cell. In the particular case of the DNA titration by peptides, peptide aliquots were added to 10 μm samples and the spectrum of LTR free of ligand was subtracted from that of the complex. This subtraction is not necessary for titrations by drugs as both RAL and TB11 are achiral molecules. Most experiments were recorded at 10 °C. Spectra were expressed as a molar CD (Δε) as a function of wavelength (λ nm). The α helix content was estimated by the relation Pα = −[Δε_222_ × 10] (Pα: percentage of α helix; Δε_222_: CD per residue at 222 nm) 41.

### Fluorescence experiments

The fluorescence anisotropy and intensity studies were recorded on a Jobin‐Yvon Fluoromax II instrument. Fluorescence anisotropy was expressed as:

$$A=\frac{I//-I⊥}{I//+I⊥},$$

where *I*//and *I*⊥ referred to parallel and perpendicular emission components. These two components were measured in L‐format. The denominator of *A* is simply the total light that would be observed if no polarizers are used. For the fluorescein reporter, maximum wavelengths from the xenon lamp (150 watts ozone‐free) were for excitation λ = 488 nm and for emission λ = 516 nm in the case of LTR34, λ = 488 nm and λ = 515 nm in the case of LTR32 and LTR32‐*GT* and λ = 488 nm and λ = 514 nm in the case LTR30. The slit for excitation width was fixed at 4 nm and emission width was 5 nm for LTR34 and 4 nm for LTR32, LTR32‐*GT* and LTR30. Samples were placed in 1 cm × 0.5 cm thermally jacketed quartz cells, controlled by a circulating bath. For each titration point, at least 10 data points were recorded at 5 °C with an integration time of 1s. In fluorescence anisotropy measurements, *I*//and *I*⊥ intensities of background solution (i.e. buffer and solute contributions) were subtracted from the sample value. Oligonucleotides were diluted to the desired concentration in 800 μL assay buffers (10 mm Na/Na_2_PO_4_, pH 6.0) with or without 5 mm Mg^2+^. Samples of, generally 10 nm oligonucleotide were titrated by the addition of aliquots of drugs or peptides. After each addition the solution was allowed to equilibrate for least 2 min. The validity of fluorescence anisotropy experiments was controlled by measuring the total fluorescence intensity in parallel to fluorescence anisotropy. As variations of fluorescence were weak, we considered that the anisotropy signal contained the desired information on complex formation. Finally, *K*_d_ (equilibrium dissociation) constants were calculated by fitting the sigmoidal curves, using graphpad prism 5 (Graphpad, Software Inc., San Diego, CA, USA) applying the nonlinear regression (curve fit) ‘Least Squares’ procedure.

In fluorescence intensity studies, fluorescence of TB11 or RAL was measured at the 20 μm concentration in 800 μL of reaction buffer. Excitation at 313 nm for TB11 and for RAL provided an emission between 325 and 500 nm, using 2 and 5 nm excitation and emission slit widths respectively. Maximal emission was measured at 410 nm for TB11 and 413 nm for RAL.

### Intercalative binding

Many intercalating drugs such as doxorubicin (or adriamycin) belonging to the anthracycline family 42 as well as the related mitoxantrone 43 continue to be extensively used in cancer treatments 44. In the intercalation mode, the chromophore is inserted in between adjacent base pairs, where it is stabilized by several types of interactions including polarization and van der Waals forces 45. Intercalation produces both an extension and left‐handed unwinding of the helix 46 and generates electrophoretic retardation of DNA, the extent of which depends on the geometry and chemical structure of the intercalator.

### Gel retardation assay

The agarose electrophoretic retardation assay was carried out as previously described 47. Briefly, TB11 and RAL were incubated at different concentrations with 250 ng of pBR322 plasmid DNA in binding buffer (20 mm HEPES pH 7.5, 5 mm DTT, 10% PEG‐4000, 10 mm MgCl_2_, 20 mm NaCl and 20 μm ZnCl_2_), in 10 μL total volume. Mixtures were incubated 30 min at 4 °C before addition of 2 μL of loading buffer (0.25% xylene cyanol, 0.25% bromophenol blue, 50% glycerol and 200 mm Tris‐HCl pH 7.5). Samples were then loaded onto 0.8% agarose gels and electrophoresed in 1× TBE for 5 h at 80 V. After electrophoresis, gels were stained in BET and visualized under UV light.

## Results

### Design and synthesis of peptides K156 and K156‐loop140

K156 is a helix stabilized peptide designed to mimic the α4 helix (residues 147–169) observed at the surface of the enzyme at the junction of the active site 36, 37. Several amino acid residues of the α4 peptide deemed unimportant for DNA recognition have been replaced by more helicogenic residues. The obtained K156 peptide now displays a backbone conformation more similar to the protein α4 helix. K156 can therefore be considered more functional and less prone to aggregation than the native α4 peptide, and its binding properties may now better reflect those of the α4 helix included in the protein. K156‐*loop140* is a hybrid peptide that extends over 33 amino acids (residue 136–168) and results from the fusion of the native flexible loop140 38, 40, 48 with the helix stabilized K156. The two peptides were synthesized by solid phase chemistry at a purity grade > 98%.

### Binding of K156‐loop140 to LTRs

#### CD spectroscopy

**Figure 2:**
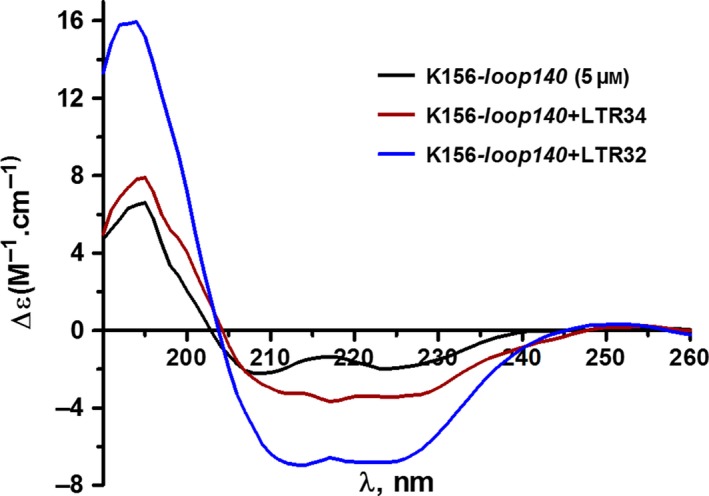
CD spectra of free DNA and DNA bound to K156‐*loop140* peptide. Spectrum of K156‐*loop140* taken alone (black line); difference spectra of complexes: [K156‐*loop140* (5 μm) + LTR34 (10 μm)]‐LTR34 (10 μm) (red line) and [K156‐*loop140* (5 μm) + LTR32 (10 μm)]‐LTR32 (10 μm) (blue line). Samples dissolved in phosphate buffer pH 6, 10 mm, and spectra were recorded at 10 °C.

The CD method is widely used to assess the secondary structures of proteins and nucleic acids and to follow their conformational changes induced by interactions and any modification in the medium 49, 50. This method is therefore well suited to the study of complexes formed between proteins and nucleic acids or between these molecules and organic ligands, such as inhibitors. In this study, we analysed the complexes of LTRs and peptides, LTRs and inhibitors, peptides and inhibitors and combinations thereof, in phosphate buffer with or without 5 mm Mg^2+^. In an earlier CD study, we have shown that the helix content of the α4 helix analogue, K156, was enhanced through its interaction with LTRs 36. The spectrum of K156*‐loop140* also displayed two negative components at ≈ 225 nm and ≈ 205 nm, and one positive at ≈ 190 nm, which was rather similar to the spectrum of K156. However, the signal intensity at 225 nm agreed with a helix content of nearly 20%, which is lower than that found for the shorter K156 37. This decreased helix average in the K156‐*loop140* was due to the incorporation of the unordered loop140 moiety beside the ordered K156 helix. We found via binding experiments that the processed LTR32 increased the helix content more than the unprocessed LTR34 (35% versus only 15%) (Fig. 2), showing that the cavity created by 5′GT3′ depletion facilitated contacts favourable to helix conversion of the peptide loop. The cleavage of 5′GT3′ allowed the peptide to enter the cavity and undergo the conformational changes desired for IN activity. According to several crystal structures, these are loop residues at the α4 helix‐loop junction which are converted into helices 20, 30, 42, 51. What are the nucleotide binding partners of the DNA cavity involved in helix induction? To answer this question, we performed CD experiments using the LTR analogues modified on their extremities. These studies indicated that in contrast to LTR34 and LTR32, neither LTR32‐*GT* nor the blunt‐ended LTR30 had increased helix content (not shown), highlighting the key contribution of the 5′AC3′ extremity of the unprocessed strand in binding the loop and setting up a functional helix. This binding did not exclude the binding of the processed strand with the peptide loop, through the liberated 3′ adenine‐OH group bound to Mg^2+^.

#### Fluorescence anisotropy

**Figure 3:**
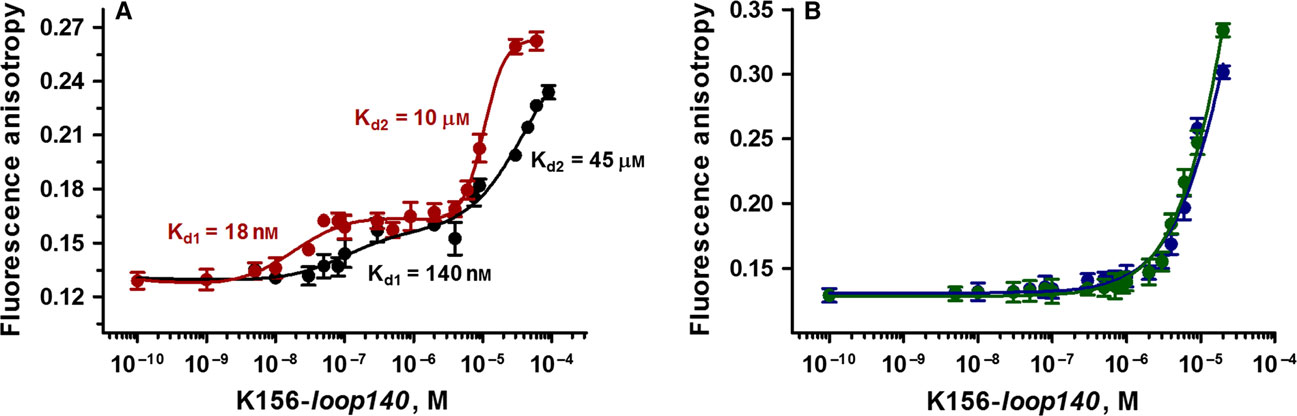
Fluorescence anisotropy titration curves: (A) LTR34 by peptide K156‐*loop140* (black line) and LTR32 by peptide K156‐*loop140* (red line); (B) LTR30 by peptide K156‐*loop140* (blue line) and LTR32‐GT by peptide K156‐*loop140* (green line). Samples were dissolved in phosphate buffer pH 6, 10 mm, and spectra were recorded at 5 °C. Note that only the titration curves of LTR34 and LTR32 are biphasic.

Fluorescence anisotropy is a highly appropriate method to assess the properties of DNA and proteins to partner together and fix ligands 37, 52. This technique provides thermodynamic parameters such as *K*_d_ values related to complex stability and stoichiometry 27. We performed the titrations by fluorescence anisotropy of unprocessed LTR34, processed LTR32 and LTR analogues with the peptide K156‐*loop140* in phosphate buffer with 5 mm Mg^2+^. With LTR34 and LTR32, we obtained biphasic curves which are typical for two distinct DNA sites (Fig. 3A). We found a *K*_d1_ of 18 nm and a *K*_d2_ of 10 μm for LTR32 and a *K*_d1_ of 140 nm and a *K*_d2_ of 45 μm for LTR34. In contrast, the titration curves of LTR32‐*GT* and blunt‐ended LTR30 were monophasic (Fig. 3B); in both cases, the *K*_d_ was in the 10 μm range and was thus similar to the *K*_d2_ values obtained with LTR32 and LTR34 standing for nonspecific binding sites.

Remarkably, the suppression of high affinity binding upon elimination of the 5′AC3′ highlights once again the importance of this step in the recognition of the 3′Pr site by IN 53, 54. The obtained *K*_d1_ values were in the range of a specific recognition of the LTR34 and LTR32 ends by the K156‐*loop140*. The greater affinity of K156‐*loop140* for LTR32 compared to LTR34 correlated with the larger amount of helix induction in the peptide by LTR32 in CD experiments. The binding of the peptide to the LTR ends and its helix stabilization through interactions with the 5′AC3′ step are linked events that agree with the picture given by the PFV intasome crystal structure 16. However, there are also metal mediated interactions, including catalytic E152, with the liberated adenine 3′OH group being located at the facing active strand extremity. The peptide loop is also able to interact with the 5′AC3′ site of unprocessed LTR34.

It is worth noting the narrow correlation between the binding values of the K156‐*loop140* peptide to LTR ends as determined by fluorescence anisotropy and the degree of helix stabilization as shown by CD. The helix stabilization is still visible in the case of the unprocessed LTR oligonucleotide, signifying that the K156*‐loop140* peptide may bind to the 5′AC3′ dinucleotide before the 3′Pr step. This could be allowed by the known large fraying affecting the end of unprocessed oligonucleotide, enabling a good fit of the terminal nucleotides on the peptide loop 16, 27. Despite a significant consumption of energy due to the reduction in entropy, the strong interactions taking place between the partners permit the formation of a stable complex.

### Binding properties of TB11 and RAL to the K156‐loop140 and K156 peptides

#### CD studies

**Figure 4:**
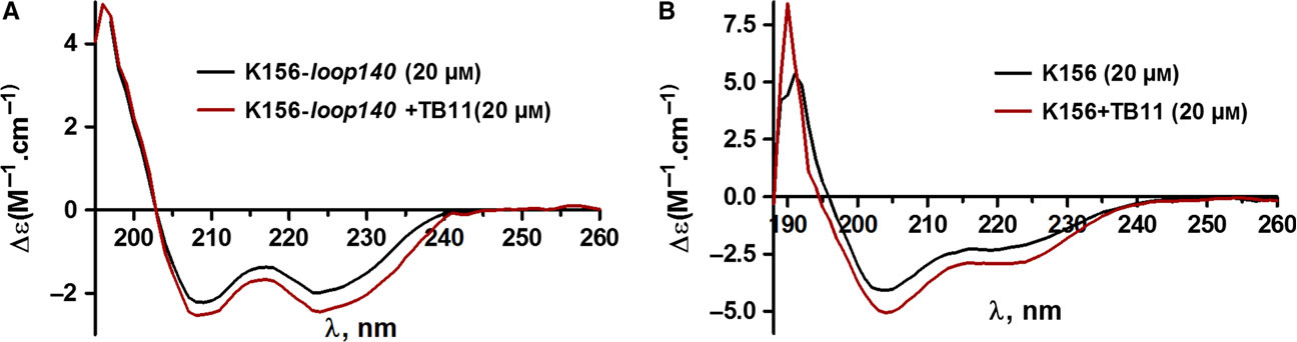
CD spectra of K156‐*loop140* and K156 peptides unbound and bound to TB11. (A) Spectra of K156‐*loop140* taken alone (black line) and of complex [K156‐*loop140 *+** TB11] (red line); (B) Spectra of K156 taken alone (black line) and of complex [K156 + TB11] (red line). Samples were dissolved in phosphate buffer pH 6, 10 mm, and spectra were recorded at 10 °C.

CD experiments provided the first evidence of important differences between the abilities of TB11 and RAL to interact with the peptides. We have shown above that the CD spectra of K156 and K156*‐loop140* were characteristic of peptides containing a rather fair amount of α helices. Upon addition of TB11 (20 μm), the helix content increased at about the same extent in K156 and K156*‐loop140* (Fig. 4A,B), suggesting that K156, that is, the α4 helix, is a target of TB11. This binding occurred only with TB11. The CD spectra of K156 and K156*‐loop140* remained unchanged upon RAL addition (not shown). Remarkably, the inability of RAL to interact with the peptides is reminiscent of that observed with the enzyme and CCD alone.

#### Fluorescence studies

**Figure 5:**
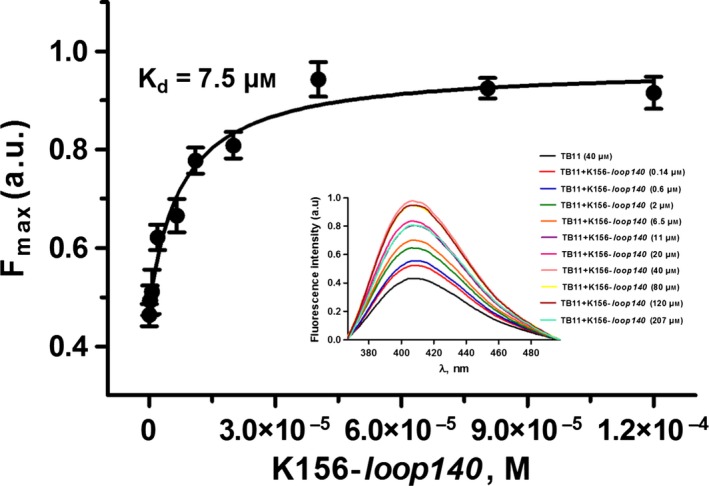
Quenching fluorescence titration curves of TB11 used as the fluorophore by K156‐*loop140*. Derived binding curve of a series of spectra of TB11 titrated with K156‐*loop140 (given in inset)* providing the *K*_d_. Samples were dissolved in phosphate buffer pH 6, 10 mm, and spectra were recorded at 5 °C.

Fluorescence experiments confirmed the CD results. According to the *K*_d_ values (7.5 and 7.75 μm), TB11 had about the same affinity for K156‐*loop140* (Fig. 5) and K156 55. This argues in favour of TB11 binding centred on K156 rather than the loop. At the same time, no binding sign was detected with RAL, which is also in agreement with the CD results 27. As mentioned above, RAL does not interact with either CCD or the enzyme alone 25, 26.

Thus, both CD and fluorescence anisotropy detected important differences in the binding properties of RAL and TB11 towards the IN peptides. The binding of TB11 to K156 was reminiscent of the binding of 5CITEP to CCD within the cocrystal structure 13. In this study, we observed four interactions between the drug and the α4 helix (Gln148, Asn155, Lys156 and Lys159). A fifth, but not unimportant, interaction involved the carboxylate group of the catalytic Glu152 residue located at the α4 helix‐loop junction in CCD. This shares a direct bifurcate hydrogen bond with the metal binding motif of 5CITEP, while in the cocrystal structure of the PFV intasome‐RAL complex, the Glu152 carboxylate group interacted with the RAL metal binding motif and the scissile phosphate via a Mg^2+^ ion. We note the involvement of several interactions of the α4 helix residues in the binding of IN to LTRs in the PFV intasome 16. One part of the 3′Pr inhibitory activity of TB11 could be assigned to IN binding, which mimics that of 5CITEP.

### Binding of TB11 and RAL to LTR ends

#### Fluorescence studies

Evidence for selective binding of IN inhibitors to LTR ends was provided by fluorescence anisotropy studies conducted with the unprocessed LTR34 and processed LTR32 oligonucleotides and two control oligonucleotides, LTR32‐*GT* and LTR30. As shown in the Material and Methods, in LTR32‐*GT,* the overhang dinucleotide 3′CA5′ was replaced by 5′GT3′ on the inactive strand. LTR30 is a blunt‐ended LTR that has been deprived of the terminal 5′GT3′/3′CA5′ base pairs. This material has already been used for the study of RAL by fluorescence anisotropy 27. Here, it served in fluorescence studies based on changes in anisotropy, shift and intensity of the emission signal 56.

#### RAL binding in the presence of Mg^2+^

Our previous fluorescence anisotropy experiments recorded in the presence of 5 mm Mg^2+^ showed that a single molecule of RAL bound to the processed LTR32 end (*K*_d_ ≈ 6 nm) 27. Regardless of the concentration of LTR32, RAL produced the same increase in fluorescence anisotropy, (from 0.13 to almost 0.145), and formed a monophasic curve in the nanomolar range. With unprocessed LTR34, the variation in fluorescence anisotropy was weaker, indicating that in this instance, a smaller fraction of RAL was fixed to the terminal base pairs 51. In addition, the affinity of RAL for LTR34 was weaker compared with that for LTR32 (*K*_d_ ≈ 20 nm versus ≈ 6 nm). At the same time, addition of RAL to the structural analogues, LTR32*‐GT* and blunt‐ended LTR 30 53, 54, had no effect on the fluorescence anisotropy spectra, even at a high drug concentration.

#### TB11 binding in the presence of Mg^2+^

**Figure 6:**
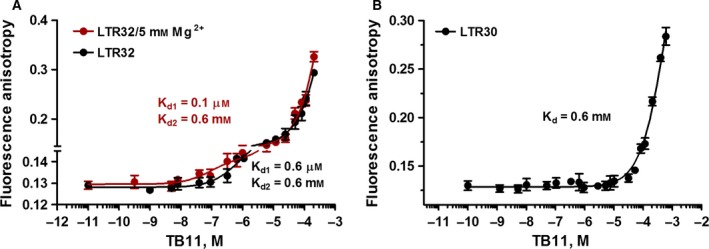
Fluorescence anisotropy titration curves of LTRs by TB11. (A) The processed LTR32 is titrated by TB11 in the presence (red line) and absence (black line) of Mg^2+^; (B) LTR30 is titrated by TB11 in the presence of Mg^2+^. Samples were dissolved in phosphate buffer pH 6, 10 mm, and spectra were recorded at 5 °C.

The titration curves for LTR34 and LTR32 by TB11 were biphasic, while we obtained monophasic curves for RAL. During the first binding of TB11, the fluorescence anisotropy increased from 0.13 up to almost 0.15 for LTR32 (Fig. 6A), but only up to 0.14 for LTR34 (not shown). Curve fitting by a nonlinear least squares procedure provided average *K*_d1_ values of ~ 0.1 μm for the binding of TB11 to LTR32 and 1.5 μm for LTR34. Thus, similar to RAL, TB11 binds more tightly to processed LTR32 than unprocessed LTR34, indicating that the depletion of the 5′GT3′ dinucleotide on the active strand facilitated the accommodation of the drug to the LTR end. Note that this was already the case for the binding of K156‐*loop140*; binding was improved by the processing. However, the double depletion of 5′GT3′ and 5′AC3′ (blunt‐ended LTR30) completely suppressed the first binding of drugs to DNA (Fig. 6B), a result that highlighted the need for the terminal 5′AC3′ dinucleotide to produce high affinity binding, which is consistent with our CD data. At the same time, these findings highlight the same requirements of the loop and the drugs for high affinity binding to viral DNA.

The binding of TB11 to the low‐affinity site was observed with the four LTR oligonucleotides independent of the presence of a high affinity site. These *K*_d_ values were in the mM range: 0.4, 0.6 and 0.6 mm for LTR34 (not shown), LTR32 (Fig. 6A) and LTR30 (Fig. 6B) respectively. The important increase in anisotropy accompanying this binding indicated that many TB11 molecules fulfil this site, thereby presenting all the features of a nonspecific binding site. We will demonstrate later that this site is exclusive to TB11 with regard to the molecular intercalation into DNA base pairs.

#### Influence of Mg^2+^ on inhibitor binding

**Figure 7:**
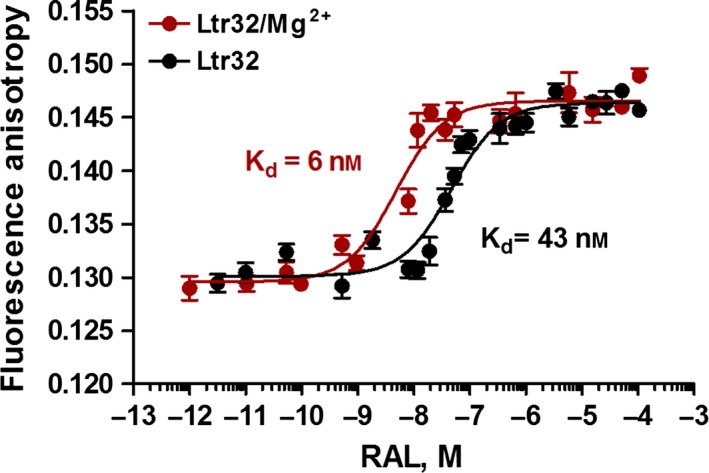
Fluorescence anisotropy titration of LTR32 by RAL. In the presence (red line) and absence (black line) of Mg^2+^. Samples were dissolved in phosphate buffer pH 6, 10 mm, and spectra were recorded at 5 °C. Estimated *K*_d_s are indicated on the curves.

The above experiments were performed in the presence of the Mg^2+^ cation 27. Divalent metal cations are known for their role not only as cofactors of catalysis but also for their impact on the local shaping and ligand binding to particular nucleotides in both the major and minor grooves of the DNA double helix 57, 58. A comparison of the *K*_d_ values obtained with and without Mg^2+^ (Fig. 7, Table 2) revealed that the divalent cation (5 mm) significantly reinforced the affinity of inhibitors for LTRs. In the absence of Mg^2+^, the *K*_d1_ values for drug binding to unprocessed LTR34 increased from 18 to 70 nm for RAL and from 1.5 to 5 μm for TB11 (not shown); for processed LTR32, it increased from 6 to 43 nm for RAL (Fig. 7) and from 0.1 to 0.6 μm for TB11 (Fig. 6A). Thus, regardless of the inhibitor, Mg^2+^ established bridging interactions between the inhibitor and the DNA substrate, and the effect was greater when LTR was processed.

**Table 2:** Recapitulation of *K*_d_ values for binding of the peptide and inhibitors to LTRs

	K156*‐loop140*	RAL	RAL/Mg	TB11/Mg	TB11
LTR32	*K*_d1_ = 18 nm *K*_d2_ = 10 μm	*K*_d_ = 43 nm	*K*_d_ = 6 nm	*K*_d1_ = 0.1 μm *K*_d2_ = 0.6 mm	*K*_d1_ = 0.6 μm *K*_d2_ = 0.6 mm
LTR34	*K*_d1_ = 140 nm *K*_d2_ = 45 μm	*K*_d_ = 70 nm	*K*_d_ = 18 nm	*K*_d1_ = 1.5 μm *K*_d2_ = 0.4 mm	*K*_d1_ = 5 μm *K*_d2_ = 0.4 mm
LTR32GT	*K*_d_ = 10 μm	–	–	–	–
LTR30	*K*_d_ = 10 μm	–	–	*K*_d_ = 0.6 mm	–

#### Role of the terminal 5′AC3′

The comparative analysis of the processed LTR32 and unprocessed LTR34 together with the LTR30 and LTR32*GT* structural analogues helped to establish the important implication of the LTR 5′AC3′ dinucleotide, alongside Mg^2+^, in the protein‐DNA complex formation. The binding of the drugs to LTR ends mimics that of the peptide loop, showing that these can compete with the peptide loop in the recognition process of 5′AC3′. Actually, numerous data reported and reviewed in the literature describe the structural and functional properties of the 5′AC3′ dinucleotide in the binding of IN and drugs 54, 59. In the recent crystal structures of the PFV intasomes 16 the role of the dinucleotide 5′AC3′ in the shaping of the loop into a helix is rather clear. The change of the backbone conformation could help the carboxylate group of the Glu152 catalytic residue to adopt a spatial orientation permitting the metal assisted interaction with the DNA site. Our studies are also consistent with previous studies performed in solution, indicating that inhibitors bind to the 5′AC3′ overhang in the intasome 54. Note, that the binding of drugs to the 5′AC3′ dinucleotide is not displayed in the crystal structure of the PFV intasome‐drug complex. In this, the 5′AC3′ dinucleotide continues to share interactions with the peptide loop, as those observed with the intasome alone 16, 22.

### Binding of RAL and TB11 to the K156‐loop140‐LTR32 complex

#### Fluorescence anisotropy

**Figure 8:**
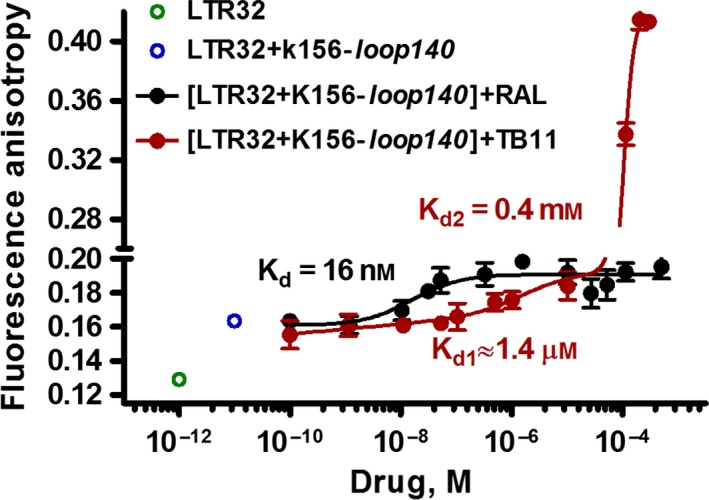
Fluorescence anisotropy titration of the LTR32 + K156‐*loop140* complex by TB11 (red line) and RAL (black line). Samples were dissolved in phosphate buffer pH 6, 10 mm, and spectra were recorded at 5 °C.

As shown above, in the presence of Mg^2+^, TB11 interacted with good affinity to the K156‐*loop140* (*K*_d_: 7.5 μm) (Fig. 5), but with a higher affinity to LTR32 (*K*_d_: 0.1 μm). RAL did not interact with the K156‐*loop140* but bound with very high affinity to LTR32 (*K*_d_: 6 nm). At the same time, 5′AC3′ was required for the binding of the K156‐*loop140* to LTRs. Thus, in all these events, the role of 5′AC3′ appears determinant, but that of the peptide loop is not as clear. We attempted to answer this question using new fluorescence anisotropy experiments where the peptide was incorporated into the complex. The titration profiles of the preformed LTR32‐K156‐*loop140* complex by TB11 and RAL showed that inhibitors did not dissociate but rather bound the complex (Fig. 8). However, the *K*_d_ value for binding of RAL to the binary complex was three times greater compared with that of LTR32 alone (16 nm versus 6 nm) (Figs 7 and 8) and rather resembles that reported for RAL binding to intasome (*K*_d_ 19 nm) 28. Comparatively, the *K*_d1_ value for TB11 binding to the binary complex was now 14 times greater than that of LTR32 alone (1.4 μm versus 0.1 μm) (Figs 6 and 8). Concerning the binding of TB11 to the low‐affinity site, this was only weakly decreased (*K*_d2_: 0.4 mm versus 0.6 mm). We still did not find a second binding site to RAL.

Overall, the addition of the peptide loop to the DNA‐inhibitor complex increased the stability of the specific complex for both RAL and TB11. The affinity of the inhibitor for the LTR‐peptide complex was weaker than that for the LTR alone. The loss of TB11 affinity was greater compared to RAL.

### TB11 intercalation into DNA base pairs

#### Gel electrophoretic retardation assay

**Figure 9:**
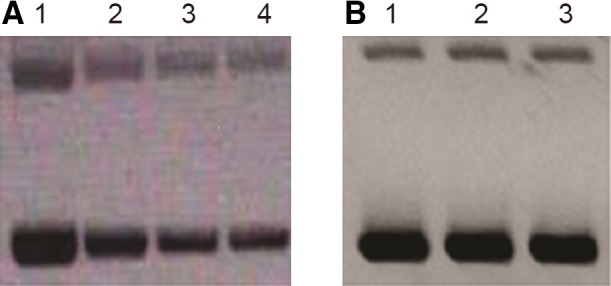
Agarose gel electrophoresis for analysis of the intercalative binding of TB11 and RAL to pBR322 plasmid DNA. Binding was assessed as described in Materials and methods. DNA helix saturation with bound molecules coincided with strongly reduced BET fluorescence, suggesting that intercalation sites for BET are occluded by the bound TB11. (A) Lane 1, 250 ng pBR322; lanes 2–4, 250 ng pBR322 and 3, 15 and 30 μm of TB11 respectively; (B) Lane 1, 250 ng pBR322; lanes 2–3, 250 ng pBR322 and 150 and 300 μm of RAL respectively.

The above fluorescence anisotropy experiments showed that the weak inhibitor TB11 had strong and weak affinity binding sites on both the unprocessed and processed LTRs, while the strong inhibitor RAL had only a very strong site. What then, is the origin of this second site? We suspect intercalation into DNA base pairs, so we performed gel electrophoretic retardation and topoisomerase I relaxation assays. These two techniques used together are well suited for detecting drug intercalation into DNA base pairs 60, 61. DNA intercalators such as anthracyclines inhibit DNA replication and are used in the chemotherapeutic treatment of cancers due to their toxic effects on tumour cells. As expected, only TB11, which displays a second binding site in the present work, retarded the migration of the pBR322 plasmid in the gel electrophoretic retardation experiments (Fig. 9), a property that is attributable to drugs that intercalate into DNA base pairs 62, 63.

The mobility of DNA decreases proportionally to the number of drug molecules inserted into DNA base pairs (Fig. 9A). The binding of TB11 coincided with a dramatic reduction in fluorescence intensity, suggesting that TB11 moves BET from its intercalating site and is therefore a good intercalator. The maximum intercalation was achieved at a drug/DNA molar ratio of 300, with the higher TB11 concentration being 30 μm. The intercalation was weakly cooperative and presented some distributive characteristics. In the case of non‐retarded and fully retarded interconverting species, we did not observe a gradual decrease in DNA bulk mobility 47. Unlike TB11, RAL did not exert a gel retardation effect, which highlights its inability to intercalate into DNA base pairs as already suggested by the absence of a second binding site for this drug in fluorescence anisotropy experiments (Fig. 9B).

The incubation of circular DNA together with topoisomerase I and unwinding ligands (intercalating drugs or some proteins) yields DNA of different superhelix densities. In fact, the torsional strain introduced by drug intercalation results in progressive DNA unwinding. The event can be demonstrated, first, by completely relaxing the BET treated DNA with an excess of topoisomerase I. Topoisomerase I removes all of the supercoils in each DNA molecule, regardless of the present degree of unwinding. Extraction of BET reintroduces supercoiling into DNA in direct proportion to the unwinding initially produced, while the control DNA, which has not been exposed to the unwinding agent, remains completely relaxed. Thus, the DNA unwinding induced by intercalating drugs is demonstrated by an increased supercoiling of final products. The results indicated that topoisomerase I completely relaxed the plasmid in the absence of BET or drug. BET at 10 μg·mL^−1^ produced a complete supercoiling of DNA, while TB11 produced a similar effect at only 3 μm (drug: DNA molar ratio 300 : 1). At the same time, RAL was completely unable to unwind the plasmid, even at a high concentration (data not shown). The above results demonstrate that the intercalation of the weak inhibitor TB11 into DNA base pairs occurs, while this effect is absent in the strong inhibitor RAL.

## Discussion

RAL and TB11 are DKA derivatives that act on HIV‐1 replication mainly by inhibiting the ST activity of IN. RAL is an INSTI that has been incorporated into the group of drugs extensively used in highly active antiretroviral treatment (HAART) 8, 64. In contrast, TB11 has been abandoned for its low bio‐availability and high toxicity (Table 1). The two drugs both bind to the 3′Pr at the LTR ends, but, whether the LTR is processed or unprocessed, RAL is always the stronger binder. Yet, the two drugs have a neat preference for the processed site. RAL is a potent inhibitor that binds with high specificity to the 3′Pr site, while TB11 is a rather poor inhibitor that has significant side effects. For the synthesis of new compounds with improved performance, we thought it interesting to learn about the physicochemical properties of the two different compounds. The comparison was mainly focused on: (a) their binding to viral LTR ends, which emerged as possible primary targets of the INSTIs 27; (b) the impact of the Mg^2+^ ions in the recognition of the 3′Pr site by drugs and the stabilization of the substrate‐drug complex; (c) the influence of the single stranded 5′AC3′ dinucleotide at the end of the processed LTR on the binding of inhibitors as this influence was not so visible in the available crystallographic studies; (d) the origin of the lower specificity and greater toxicity of TB11 compared with RAL; and (e) the good correlation between the affinity of the drug for the processed LTR bound to Mg^2+^ and their IN inhibition efficacy. Such a dependence of IN inhibition versus affinity for processed LTR constitutes a strong argument in favour of viral DNA ends as a primary site of drug interaction. Obviously, the 3′ processed LTR‐drug complex does not preclude the formation of a ternary complex with the enzyme and the induction of resistance mutations by the drug.

### Binding of the catalytic loop and α4 helix to the substrate DNAs

The binding affinity of the K156‐*loop140* peptide (peptide mimicking the stabilized α4 helix connected to the catalytic loop) to the LTR ends is similar to that of inhibitors. In both cases, the binding was tighter with processed LTR than unprocessed LTR, and it was suppressed when the AC5′/GT3′ was removed (blunt‐ended LTR30). Upon binding to the LTR ends, the K156‐*loop140* peptide undergoes a helix conversion that mainly affects the flexible loop. The phenomenon is more pronounced when the binding partner is the processed LTR32 rather than the unprocessed LTR34. Thus, there is a clear correlation between binding and helix stabilization; the stronger the peptide affinity for the DNA substrate, the greater the peptide helix stabilization. This result is consistent with the crystallographic data on the CCD and intasome structures found in the literature 16, 38, which indicate that there are residues converting into helices located in between the N terminus of the α4 helix and the end of the catalytic loop. Actually, the stabilization into a helix by a peptide through specific interactions with DNA is not an uncommon event and may result in a conformation allowing the stimulation of a biological response 65. Here, the helix stabilization within the catalytic loop at the junction with the α helix is functionally important as it properly orients the carboxylic group of the Glu152 side chain to ligate a Mg^2+^ cation (‘B’ metal ion in 22, 66). Obviously, this implicates other structural and dynamic factors that are difficult to apprehend, including the formation of a hydrogen bonding network with water molecules and monovalent cations 67.

### Role of the 5′AC3′ dinucleotide

The results provided by the four LTR oligonucleotides (LTR34, LTR32, LTR32‐*GT* and LTR30) indicated that the 5′AC3′ dinucleotide at the LTR34 and LTR32 extremity is involved in the binding of both the K156‐*loop140* peptide and inhibitors. This is especially true in the case of the processed LTR32, where access to the binding site was easier than the unprocessed LTR34, although the latter has favourable base fraying at its end. Indeed, our results confirm the prominent role of 5′AC3′ in the association of IN to DNA, as reported in several biochemical and spectroscopic papers 27, 54, 68 as well as in the recently published X‐ray crystallographic data provided by the analysis of PFV intasome complexes 16.

### On the origin of Mg^2+^ ions in the complexes and their role in the specific binding and cleavage of substrates

**Figure 10:**
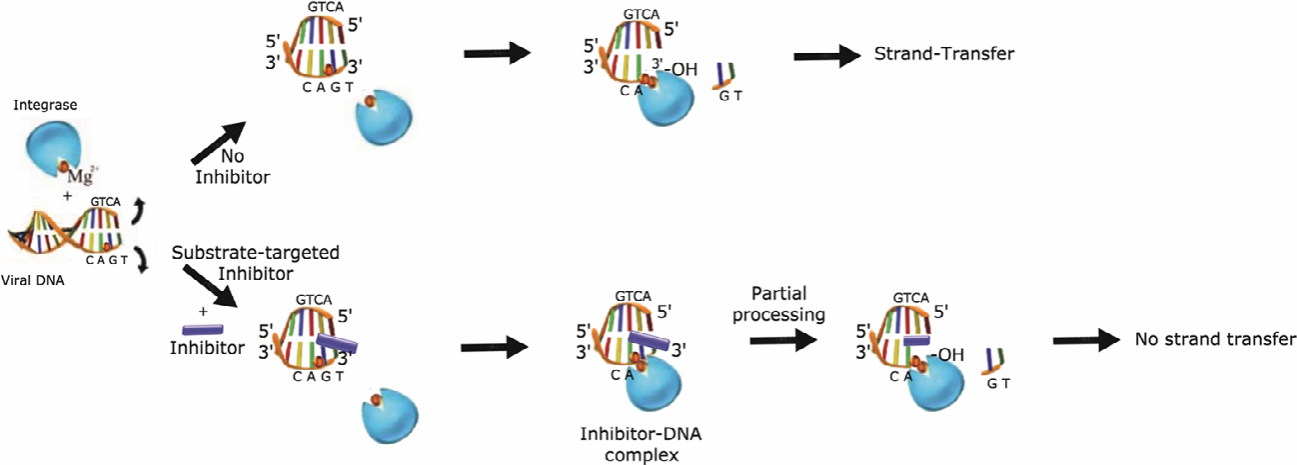
Scheme illustrating a possible inhibition mechanism of IN by drugs: (Top), the processing of LTR by IN. We assume that one Mg^2+^ ion is brought in by the protein and the other by the DNA substrate; (Bottom), the binding of the inhibitor (RAL) to unprocessed LTR (partial inhibition of processing) and to processed LTR (strong strand transfer inhibition).

Our results emphasize an important participation of Mg^2+^ ions in stabilizing the binding of inhibitors to LTR ends (Fig. 10). In fact, the X‐ray crystallographic data demonstrated the presence of a pair of divalent metal ions in the binary DNA‐IN complexes 16 and the ternary complex DNA‐IN‐inhibitors, but the latter tell us nothing about the energy of binding. Conversely, in most of the crystal structures of unbound INs reported to date, the active site contained a single metal ion. However, for both HIV‐1 and PFV, no crystal structures of the entire enzyme alone are available. The catalytic site of HIV‐1 IN CCD crystal structures 38, 40, 48, 69 shows a single divalent metal (‘A’ metal ion) chelated to the Asp 64 and 116 side chains, while the carboxylic group of the Glu 152 is always free of interaction. The crystal structures of the bi‐domain proteins, Nter‐CCD 70 and CCD‐Cter 71, provided a similar result and do not clarify the source of the second cation in the DNA‐IN and DNA‐IN‐drug complexes.

According to a molecular dynamics calculation, the second Mg^2+^ ion (‘B’) could be brought into the complex with the enzyme by the processing site of the viral DNA 72. The crystal structures of naked oligonucleotides 31, 73 and the molecular dynamics calculations based on recent crystal structures 33 agree with the above results. They provide evidence for a marked binding preference of Mg^2+^ to purine–purine dinucleotide sequences such 5′AG3′, the latter offering the best electrostatic environment to recruit divalent metal cations 31, 74. In the viral LTR substrate, 5′AG3′ coincidentally corresponds to the cleavage site and could capture a Mg^2+^ ion. This, so‐called ‘A’ would be pre‐positioned on the cleavage site. From this position, it could contribute to the recognition and cutting of the scissile phosphodiester linkage by the enzyme active site bound to the Mg^2+^ ion ‘B’ (Fig. 10). Notably, the processing site of the cleavable 5′AG3′ dinucleotide is included in a 5′CAG3′ trinucleotide. The cytosine phosphate of such a triad has been shown to participate in bridging interactions with divalent metal cations 33. It is certainly no accident that the cleavage site 5′AG3′ and the highly conserved dinucleotide 5′CA3′ are combined within the processing site to make it highly specific. Moreover, the 5′CA3′ step is extremely malleable and may help the 5′AG3′ step to accommodate its phosphate bond in a cleavable conformation within the IN active site 75, 76, 77, 78. In fact, the 5′CA3′ step is often found in DNA substrates, where it could pilot the cleavage of the connected steps by enzymes belonging to retroviruses, transposable phage Mu and also transposable elements 55, 68, 78, 79.

Moreover, the fraying that is common in the last two base pairs of DNA can be increased at the HIV‐1 LTR ends by the motions affecting the flexible 5′CA3′ dinucleotide 68, thereby explaining why drugs also bind to unprocessed LTRs.

To summarize, our results suggest that the binding of one Mg^2+^ ion to the 5′CAG3′ triad could make a particular scaffold to ensure a specific recognition and electrostatic fit by the enzyme or the drug. Thus, in the intasome complexes, one Mg^2+^ ion could be supplied by the enzyme active site and the other by the DNA cleavage site. The ability of the DNA duplex end to separate into two strands 80, 81 permits the cleavage site to adopt the in‐line geometry required for bond cutting 82. Thus, the base pair fraying is certainly an important factor permitting the best fit of inhibitors on the active strand of the cleavage site. Obviously, the deletion of the 5′GT3′ by the 3′Pr reaction permits a better Mg^2+^ assisted capture of the drug by the liberated 5′AC3′ dinucleotide strand and the adenine‐3′OH group 16, 83.

### A relationship between drug binding to processed LTR and ST inhibition

X‐ray studies 16, 22 have shown that apart from metal assisted interactions with the DNA substrate, inhibitors also share direct interactions with DNA, thus enforcing the stability of the DNA‐drug complex. RAL and its antiviral congeners use their halogenated aromatic ring to make contacts with the highly conserved 5′CA/TG nucleotides, that is, the adenine phosphate and the cytosine base of the conserved 5′CA3′, as well as the guanine of the facing 5′TG3′ 16, 27, 84. These interactions contribute to the good correlation observed between the affinity of inhibitors for the processed LTR32 and ST inhibition, giving credence to the idea that the DNA substrate is the drug target. Such a mechanism of inhibitors first binding the substrate rather than the enzyme is scarce in the literature. The only examples concern protease substrates 85, 86, 87.

### The extra binding sites and the side effects of TB11

In addition to its interaction on the 3′Pr sites at LTR ends, the TB11 molecule has other binding sites including: (a) the intercalation into DNA base pairs as revealed by both fluorescence spectroscopy, gel electrophoretic retardation assay and plasmid unwinding assay, and (b) interactions with peptides derived from the enzyme active site. The fluorescence variation induced by the TB11 intercalation into DNA base pairs (low‐affinity site) indicated a nearest neighbour exclusion mechanism 88. The obtained *K*_d_ value (10^−4^ to 10^−5^ m) agrees with the TB11 concentration required to produce the unwinding of supercoiled DNA in gel migration assays. Of note, the TB11 *K*_d_ was similar to those generally obtained with the well‐known antitumour drugs anthracyclines and acridines. These drugs are poisons targeting the topoisomerase II enzyme 89 and provoke the death of tumour cells but also healthy cells. Thus, intercalation into DNA base pairs is an event that can contribute to the toxicity of TB11.

In addition, the binding of a fraction of TB11 to the α4 helix and to the loop140 belonging to the enzyme catalytic site in the enzyme tetramer could be the foundation of the 3′Pr inhibitory activity of this compound. However, TB11 could also bind to the α4 helix and loop 140 at the surface of the two nonfunctional monomers of the tetramer. This binding is ineffective for IN inhibition, but it diminishes the number of TB11 molecules available for binding to the inhibiting site.

## Conclusion

The results presented here, comparing the efficient RAL with the weakly efficient TB11 confirm the idea that the viral DNA substrate is the primary binding site of INSTIs (Fig. 10) 27. This is strongly supported by the finding of a good correlation between the binding affinity of these inhibitors for the processed viral DNA and their efficacy towards the ST inhibition. In fact, successful antiviral drugs such as RAL optimally fit the cavity opened by 3′Pr reaction at the viral DNA end, where they share strong electrostatic interactions with the Mg^2+^ ions and van der Waals forces with the DNA nucleotide atoms, essential for the success of the ST reaction. Results prove that the 3′Pr‐Mg^2+^‐site complex is in itself a highly specific binding site for RAL and efficient INSTIs, especially as a cavity similar to that left open by the 5′GT3′ deletion at the viral DNA end, certainly does not exist away from the ends of DNA and cannot be a competitive site. In comparison, the smaller TB11 is unable to fully occupy this space and make all the interactions of the 3′Pr site. The issue of the origin of the metal cofactors (‘A’ and ‘B’), found at the IN‐LTR interface of the intasome crystal 16 is also interesting. There is no doubt that the ‘A’ ion which interacts with the carboxylate group of the crucial Asp 64 and Asp 116 residues in some crystal structures of the DNA‐free CCD, is brought in the IN‐DNA and IN‐DNA‐drug complexes by the enzyme active site itself. Concerning the origin of the ‘B’ ion, we assume, in agreement with the hypothesis of Lins *et al*. 72, that it is carried in the intasome by the DNA substrate itself. When the DNA substrate is the unprocessed LTR, the ‘B’ ion could be pre‐positioned on the 5′CAG3′ triad of the active strand, ready to capture INSTI drugs, through mainly electrostatic interactions.

## Author contributions

Conceived and designed the experiments: FFA, ZH, LZ, RGM, SF. Performed the experiments: FFA, ZH. Analysed the data: FFA, ZH, SA, LZ, RGM, SF. Contributed reagents/materials/analysis tools: LZ, SF. Wrote the paper: FFA, ZH, RGM, SF.
